# Beyond the Individual: A Data-Driven Approach to Protect Kidneys from Environmental Change

**DOI:** 10.34133/hds.0405

**Published:** 2026-07-03

**Authors:** Feifei Zhang, Luxia Zhang

**Affiliations:** ^1^ Center for Digital Health and Artificial Intelligence, Peking University First Hospital, Beijing, China.; ^2^ Beijing Key Laboratory of Research, Development and Translational Application of Multimodal Intelligent Diagnosis and Treatment System, Beijing, China.; ^3^ Renal Division, Department of Medicine, Peking University First Hospital, Beijing, China.; ^4^ National Institute of Health Data Science at Peking University, Peking University Health Science Center, Beijing, China.; ^5^ State Key Laboratory of Vascular Homeostasis and Remodeling, Peking University, Beijing, China.

The escalating global challenge of environmental change—including rising temperatures, worsening air pollution, water scarcity, and expanding chemical contamination—is profoundly reshaping the epidemiology of kidney disease worldwide. The kidneys, highly sensitive to heat, hypovolemia, and environmental toxins, act as sentinel organs for environmental stress, with health impacts ranging from acute kidney injury (AKI) and nephrolithiasis to accelerated chronic kidney disease (CKD) progression and emerging forms of endemic CKD of unknown cause (CKDu) [1,2]. Responding to this challenge requires 2 complementary strategies: mitigation (addressing root causes through climate governance and emission reduction) and adaptation (protecting vulnerable populations through clinical and public health interventions). Advances in data science—particularly causal inference and artificial intelligence (AI)—provide the opportunity to translate these insights into actionable clinical and public health responses and enforceable policy (Fig. 1). In this perspective, we outline how integrated data approaches can bridge the gap between knowledge and action and provide a road map for research-to-policy translation that advances both mitigation and adaptation goals. Throughout, “environment” refers to the external exposome (i.e., multisource environmental exposures) including ambient heat and heat waves, air pollution, water and chemical contamination, and the acute disruptions of natural disasters [3,4].

**Fig. 1.**
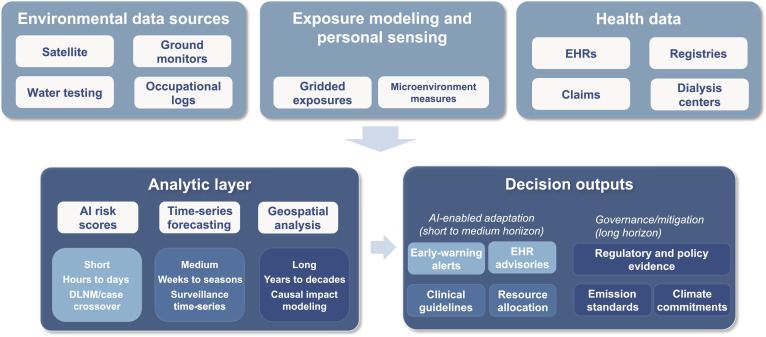
Data-to-policy pipeline. EHRs, electronic health records; AI, artificial intelligence; DLNM, distributed lag nonlinear model.

## Kidneys as a Sentinel Organ in a Changing Planet

**Fig. 2.**
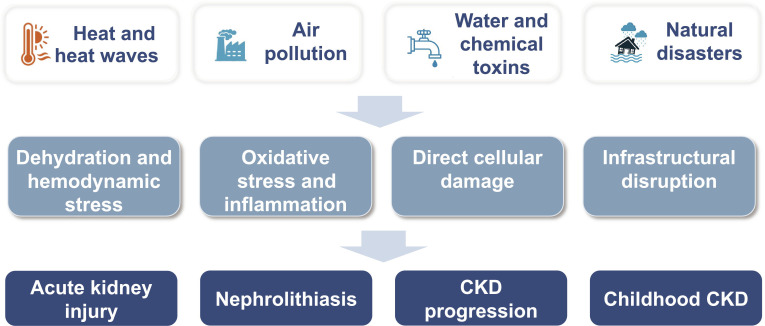
Environmental risks to kidney health. CKD, chronic kidney disease.

Kidney physiology concentrates blood-borne substances and tightly regulates volume and electrolytes, making the organ uniquely vulnerable to environmental stressors. Environmental threats to kidney health operate through multiple pathways (Fig. 2). First, thermal and hemodynamic stress: ambient heat and heat waves drive dehydration and rhabdomyolysis, leading to increases in AKI, nephrolithiasis, accelerated CKD progression, and geographically clustered forms such as CKDu [1]. Second, pollutant chemical exposure: long-term exposure to fine particulate matter (PM_2.5_), heavy metals, pesticides, and novel contaminants such as micro- and nanoplastics reduces the estimated glomerular filtration rate (eGFR) and promotes albuminuria and CKD incidence, primarily through oxidative stress, inflammation, and direct cellular damage [5–8]; early-life exposure to endocrine-disrupting chemicals further contributes to congenital kidney abnormalities, a leading cause of childhood CKD [9]. Third, physical and infrastructural disruption: natural disasters operate through a fundamentally distinct etiological pathway—crush injuries precipitate AKI through rhabdomyolysis-independent traumatic mechanisms while simultaneously interrupting life-sustaining dialysis care [10].

Recognizing kidneys as environmental sentinels reframes nephrology: from organ-level descriptions to population-level priorities for mitigation and clinical adaptation. Major guideline bodies (e.g., Kidney Disease: Improving Global Outcomes [KDIGO]) and European bodies (notably the European Renal Association) now explicitly identify climate change, air pollution, and healthcare sustainability as priorities for kidney health, although environmental risks remain incompletely integrated into routine, general-population guidance [11]. Our 2025 CK-NET Annual Data Report adds to this agenda by presenting environmental analyses and practical recommendations for embedding environmental metrics into CKD surveillance and care pathways [12]. Data science offers the methodological tool kit to move environmental nephrology from descriptive associations toward actionable decision support for clinicians, public health planners, and policymakers.

## The Need for Governance: Guiding Environmental Mitigation Policy with Causation Inference

The long-term burden of environment-related kidney disease exhibits marked inequities: risks concentrate in low-resource countries, marginalized communities, and vulnerable occupational groups. To address these inequalities and curb future disease burden, data-driven environmental risk assessments over years to decades are essential to inform climate governance and regulation, such as emission reduction. Traditional ecological studies have been instrumental in sounding the alarm—for example, that every 1 °C increase in temperature is associated with an approximate 1% rise in kidney-related morbidity [13]—but population-level associations often fail to account for individual confounders such as socioeconomic status, comorbidity, and access to care. The causal inference paradigm provides the rigorous, long-horizon evidence base required to evaluate the real-world impacts of policies (e.g., air quality standards, industrial emission limits, and workplace heat rules) on kidney health [14]. Importantly, nephrology-specific data can uniquely strengthen general environmental regulation: population-level kidney health metrics—including eGFR trajectory surveillance, AKI hospitalization rates, and CKDu incidence—can serve as renal-specific endpoints within regulatory impact assessments, supplementing the cardiovascular and respiratory metrics traditionally used in air quality standard reviews. For instance, using quasi-experimental designs and difference-in-differences methods, researchers can assess the real-world impact of specific regulations (like the Clean Air Act) on kidney health outcomes including GFR, blood urea nitrogen, and uric acid [15]. Another study leveraging an instrumental variable model provided robust, causal evidence that long-term PM_2.5_ exposure reduction contributes to the preservation of kidney function [16]. Advanced methods such as marginal structural models, g-estimation, and target trial emulation further address time-varying confounders and feedback loops that standard quasi-experimental designs may miss [17,18].

Beyond establishing causation, causal methods elucidate biological pathways from exposure to disease. Causal mediation analysis has quantified oxidative stress and inflammatory biomarkers as important mediators of heavy metal and PM_2.5_ effects on eGFR, identifying actionable intervention points beyond the exposure itself [19,20]. Multi-omics integration—encompassing epigenetic clocks and methylation risk scores that capture cumulative environmental effects on the biological aging of the kidney, with mendelian randomization providing causal anchoring of molecular findings—serves 2 translational roles: strengthening the biological plausibility criterion required in regulatory risk assessments and generating population-deployable biomarkers that bridge high-dimensional molecular data and the population-level metrics used in environmental regulation [21–23]. These molecular markers of biological aging may offer more stable, long-term risk scores than transient clinical markers, better aligning adaptation strategies with the chronic, cumulative nature of environmental kidney damage. Together, this mechanistic tool kit moves multi-omics from a descriptive research tool toward a functional component of the evidence base for environmental kidney health policy.

## The Urgency of Adaptation: Empowering Individualized Precision Medicine with AI

While governance and mitigation efforts are essential, they are often slow to manifest benefits. AI is a core enabling technology for precision adaptation. AI models—including random forests, gradient-boosted machines, and ensemble learners—can synthesize exposome, clinical histories, medication profiles, and biomarkers to produce individualized risk probabilities [24]. Pioneering work in pediatric cohorts has demonstrated the feasibility of creating “environmental-clinical risk scores” that harmonize disparate exposures into a single predictive metric [25]. Applied to nephrology, such models could identify the specific diabetic CKD patient in an urban heat island most likely to be hospitalized during a heat wave, enabling targeted preemptive measures (e.g., outreach to patients on nephrotoxic medications, intensified home monitoring for transplant recipients, or temporary dialytic capacity expansion). Early inpatient AI classifiers can also predict which heatstroke admissions will progress to AKI, guiding escalation of care in the first 24 h [26].

Beyond short-horizon individualized risk prediction, AI can also model complex gene–environment (G × E) interactions to explain why individuals respond differently to the same exposure. Genetic factors—for example, the APOL1 high-risk genotype common in individuals of African ancestry—substantially modify CKD risk, but penetrance is often incomplete, suggesting that environmental “second hits” (e.g., infections, pollution, and heat exposure) may trigger disease [27]. AI algorithms are uniquely capable of modeling these nonlinear, high-dimensional interactions and quantifying how genetic risk interacts with environmental exposures to produce disease. This enables truly personalized prevention strategies and helps prioritize interventions across environmentally susceptible subpopulations, including those with genetic susceptibility, children, occupational workers, older adults (facing compounded heat–AKI risk from reduced thermoregulatory reserve and polypharmacy), individuals with cognitive impairment, and those with intersecting social determinants of health. To support nephrologist adoption, such models should incorporate explainability methods—such as Shapley additive explanations values—ensuring that risk outputs are transparent and actionable at the point of care [28].

## Forecasting and Early Warning: Building System-Level Adaptation with AI

Beyond individual alerts, AI enables system-level preparedness across the full temporal spectrum of environmental kidney risk. Reactive healthcare systems are ill-equipped for the acute shocks of a changing climate; data science empowers a proactive stance through predictive forecasting. Environmental exposures are inherently dynamic, and different temporal scales demand different modeling approaches and decision-maker audiences (Fig. 1). At the event level, distributed lag nonlinear models and case-crossover designs have characterized the lag structure between short-term exposure to ambient heat wave or air pollution and kidney disease outcomes—for example, showing that AKI risk typically peaks 48 h after extreme heat exposure, a predictable window that early-warning systems must exploit [29]. Near-real-time meteorological and air quality feeds linked to electronic health records (EHRs) and dialysis registries enable operational AKI surge prediction, triggering tiered public health responses such as hydration messaging, clinic staffing adjustments, medication safety alerts, and targeted outreach to high-risk patients. At the medium term (weeks to seasons), surveillance of CKD exacerbations and seasonal prescribing of threshold adjustments inform clinical guidelines and resource planning. At the long horizon (years to decades), quasi-experimental and causal inference studies quantify the kidney health impact of regulatory interventions—complementing the governance section above and closing the loop between data, policy, and measurable population-level outcomes.

Geospatial analytics further support health-system resilience planning [30]. Kidney care, particularly dialysis, is acutely vulnerable to environmental disruption, being dependent on stable power, water, and transport networks. AI-generated vulnerability maps overlaying floodplain maps, wildfire risk zones, and dialysis center locations can identify facilities and populations at greatest risk [31] and guide interventions such as staggered scheduling, securing of backup power, pre-stocking of consumables, and the strategic positioning of mobile dialysis units. Network simulations of patient flow can optimize alternate routing during disruptions. These measures materially improve resilience and reduce morbidity and mortality during disasters.

## From Evidence to Guidelines and Policy: Linked Mitigation–Adaptation Pathways

Data bridges clinical practice and policy. Causal inference that emission reduction improves renal outcomes underpins stricter air quality and chemical-safety standards. Simultaneously, predictive systems can be embedded in future KDIGO guidelines: climate-adjusted thresholds for diuretic dosing, EHR-integrated alerts for nephrotoxin use during extreme heat—designed within clinical decision support stewardship frameworks using tiered severity and patient-context filters to minimize alert fatigue—and protocols for dialysis center preparedness. Embedding kidney health metrics (heat-related AKI incidence and CKDu prevalence) into national climate adaptation reports and Paris Agreement commitments would create accountability and align mitigation targets with measurable health outcomes.

## Conclusion: Linked Strategies for Durable Kidney Health

Environmental threats to kidney health are real and rising. A dual strategy—rigorous causal evidence to drive mitigation, paired with AI-enabled precision adaptation—offers the most efficient path to reduce immediate harms and curb long-term burden. Realizing this road map equitably demands confronting a shared data challenge at multiple levels: breaking down silos between environmental and health systems, addressing the infrastructure gap in low- and middle-income countries where CKD burden is highest, and building resilience to sensor dropout and sparse regional data—areas where federated learning, ensemble imputation, and transfer learning show promise. The path forward also includes investing in improved exposure measurements and navigating ethical and governance challenges. Translating the “sentinel organ” concept into practice requires defining measurable thresholds—such as population-level eGFR decline rates or AKI admission surges—to trigger standardized monitoring and public health responses, and this remains a key research priority. The evidence of environmental threat to kidney health is irrefutable, and the cost of inaction is far greater. The nephrology community, in collaboration with data scientists, public health experts, and policymakers, must now lead the charge.
